# Innovations in Simulation: Scaffolding a Triage Experience

**DOI:** 10.1111/jmwh.70067

**Published:** 2025-12-10

**Authors:** Kathryn McDevitt, Rhea Freeman‐Williams, Elizabeth “Beth” Robson, Catherine Daily, Kathryn Atkin

**Affiliations:** ^1^ Georgetown University School of Nursing Washington District of Columbia; ^2^ University of Maryland School of Nursing Baltimore Maryland

**Keywords:** ambulatory care, interprofessional collaboration, midwifery education, obstetric triage, pregnancy complications, simulation

## Abstract

Certified midwives, certified nurse‐midwives, and women's health nurse practitioners (WHNPs) play a vital role in triaging pregnant patients in the ambulatory and inpatient settings. Demonstrating proficiency with this skill set requires time and experience. In an effort to provide students the opportunity to develop their triage skills, a triage activity was designed and implemented at a large, distance‐based hybrid nurse‐midwifery and WHNP program. The aim of this simulation was to help students integrate knowledge attained from their second specialty ambulatory care course with an online simulation activity designed to provide practical hands‐on application. The 14‐week ambulatory care course work focused on complex pregnancy care with weekly meetings in a live virtual classroom and included a clinical companion course. On the last day of the term, students were assigned to work in small groups with different rotating roles. Faculty assumed the role of the patient while using an interactive PowerPoint to bring the simulation to life. Students had the opportunity to rotate through different roles—nurse, provider, and consultant, and simulated triaging pregnant patients via telehealth, in the office, and hospital setting. After each scenario, faculty debriefed with the students about the approach and content. The intention was to offer a safe space to hone challenging skills, practice delegation of tasks, and sort through complex triage content. This article describes the implementation of this virtual triage simulation activity as a blueprint for other programs to adopt similar innovative educational experiences.

## INTRODUCTION

The role of certified nurse‐midwives (CNMs), certified midwives (CMs), and women's health nurse practitioners (WHNPs) in the provision of triage services for pregnant people is well established and supported by the American College of Nurse‐Midwives (ACNM), the American College of Obstetricians & Gynecologists, and the National Association of Nurse Practitioners in Women's Health (NPWH).^1^, ^2^, ^3^ Although the skills to provide triage services are complex and multifaceted, preparation for this role is not well established in graduate programs, with much of the training occurring in the clinical setting with preceptor oversight. In addition, demonstrating competency in triage skills or completion of triage hours are not prerequisites for graduation per accreditation standards established by Accreditation Commission for Midwifery Education or Commission on Collegiate Nursing Education.^4^, ^5^ Lack of clinical sites and available preceptors further limit learning opportunities for students, yet managing perinatal triage is an expected part of full scope practice for WHNPs and CNMs/CMs. In response, faculty at Georgetown University Berkley School of Nursing, a large distanced‐based nurse‐midwifery (NM) and WHNP dual track and WHNP single track education program, created a virtual synchronous triage simulation activity. The program values the flipped learning classroom approach and emphasizes care of the whole person, *cura personalis*, while integrating health equity throughout the curriculum, aligning with ACNM's hallmarks of care and NPWH's client‐centered care competencies.^6^, ^7^ Throughout the program students engage in live, synchronous weekly meetings while having access to asynchronous content that they complete on their own time. These materials include readings, online modules, videos, and assignments to help prepare them for the live, synchronous classes. The primary aim of this innovation was to increase students’ readiness to provide triage services and standardize the learning experience.

  

Continuing education (CE) is available for this article. To obtain CE online, please visit http://www.jmwhce.org. A CE form that includes the test questions is available in the print edition of this issue.

### Course and Activity Background

During their second term of specialty courses, students enrolled in the NM/WHNP and WHNP programs are required to take *Complex Pregnancy Care* along with a clinical companion course. This course is preceded by a foundational pregnancy care course, which covers routine prenatal care and directly precedes the intrapartum clinical and didactic courses for the dual track students and integration clinical and didactic courses for the WHNP students. The *Complex Pregnancy Care* course focuses on the management of pregnant people with complex pregnancy conditions with weekly sessions. From the beginning of the term, students are provided with asynchronous content on the process of triaging pregnant patients, assigned readings, and a review of the more common and high‐risk perinatal ambulatory and intrapartum triage presentations.

**QUICK POINTS**

✦ Providing triage services to pregnant individuals requires a complex and multifaceted set of skills.
✦ Opportunities for clinical experiences in perinatal triage settings are limited and require innovative strategies for students to receive standardized education and demonstrate competency in their skills.
✦ The triage simulation activity bridges the learning gap from theory to practice and creates an effective way for students to improve knowledge, practice delegation, communication, and consultation while gaining self‐confidence.

This innovative educational activity was built upon a didactic breakout activity with different perinatal triage scenarios. It transformed into an immersive simulation, providing students with the experience of evaluating pregnant people with common high acuity clinical presentations across a variety of settings. Although simulation is a well‐established educational strategy within the program, this novel approach to teaching about the process of triage created an opportunity for students to practice clinical reasoning, prioritization and decision‐making in a low‐stakes environment.

The majority of pregnant people who present to the hospital are initially evaluated in a designated area during a process as well as a location commonly referred to as triage. Patients are most commonly seen for labor evaluation; however, other common reasons include evaluation of preterm labor, vaginal bleeding, decreased fetal movement, abdominal or pelvic pain, and signs and symptoms of preeclampsia.^8^ During the simulation, the triage process includes an initial timely assessment by the student after receiving sign out from the office‐based medical assistant or the advanced practice clinician leaving their shift. During the simulation, another student assumes the role of registered nurse (RN) who is given a script and can then provide additional patient information as requested. The student must determine prioritization of patient evaluation based on the clinical presentation and objective data as provided by the RN. The next steps may include delegation, immediate evaluation, consultation, or transfer of high‐risk patients to another facility that is equipped to provide the necessary level of care.^2^

Students have reported challenges in retaining and applying the volume of information especially within the triage setting. This is likely linked to the unpredictability of patient diversity in the clinical setting as well as complex patient presentations. To help with this problem, faculty created a triage virtual simulation experience for students enrolled in this course using Kolb's experiential learning theory and the flipped learning theoretical frameworks.

### Theoretical Frameworks

To provide a conducive learning environment for the students, this activity was structured as an ungraded formative simulation experience based on Kolb's experiential learning theory.^9^ Kolb's experiential learning theory asserts learning occurs through a transformational experience with a 4‐phase learning cycle that includes a concrete experience, reflective observation, abstract conceptualization, and active experimentation.^9^ Well‐designed high‐fidelity simulation experiences have been shown to bridge the learning gap from theory to practice and provide a safe learning environment for students enrolled in advanced practice nursing education programs.^10^ Kolb's experiential learning theory is well suited for designing successful simulation based learning activities.^9^

This activity was structured using a flipped learning model to be consistent with the curricular expectations of the program. Flipped learning is grounded in constructivism learning theories, which affirms that students learn new information through making connection points with their existing information, therefore extending their comprehension of the new material.^11^ Flipped learning in graduate education increases critical thinking skills, problem solving, student engagement, and confidence.^12^ The 4 pillars of flipped learning include fostering flexible environments, providing intentional content, a diverse learning culture, and facilitation by prepared faculty.^11^ Within this framework, graduate students complete the assigned content prior to meeting. During the weekly, live sessions, students apply, analyze, and evaluate the content with structured activities.^12^

Virtual simulation, rooted in the Kolb's experiential learning theory, focuses on the interactive multidimensional application of the learned material and is an effective way to improve knowledge and student satisfaction in nursing education.^9^, ^13^ When integrated into the flipped learning model, virtual simulation has been shown to improve time management, knowledge attainment with application, and examination performance in health care education.^14^, ^15^ These complementary educational modalities increase student self‐confidence and critical thinking and improve time‐management skills for midwifery and other advanced practice students.^16^

## CURRICULUM IMPLEMENTATION

The interactive triage simulation was the culmination of the *Complex Pregnancy Care* course. Students and faculty worked in small groups via breakout rooms using Zoom. It was an opportunity for students to apply the skills they learned throughout the term in an interactive way. The small group format with rotating assigned roles (provider, nurse, and consultant) allowed students to engage in both active and observational learning while providing concrete educational experiences from differing perspectives. Scripts written by faculty based on identified learning gaps throughout the term are intended to challenge students but not enough to negatively impact learning. Postsimulation debriefings enabled students to engage in reflective observation and form abstract conceptualizations.

The faculty's role as patient and debriefer created an environment that supported the social constructivism zone of proximal development concept, where students can perform more challenging tasks with faculty guidance. The goal was to create a *sweet spot* to optimize learning by providing students with the opportunity to demonstrate knowledge and skills in areas in which they function autonomously but also have the ability to consult when certain management may extend beyond their knowledge or comfort level. This scaffolded approach with immediate debriefing allowed students to shape their understanding while ensuring alignment with learning objectives.^9^

## TRIAGE SIMULATION ACTIVITY

During the final week of the course, students were provided with learning objectives through the Canvas online learning management system. This content identified specific health complications in pregnancy that had not been previously addressed in the weekly content as preparation for the upcoming simulation activity scheduled on the last day of the course. The complications included epilepsy, asthma, cardiac disease (peripartum cardiomyopathy and valvular dysfunction), gastrointestinal disease (cholelithiasis, cholecystitis, appendicitis, acute liver failure, and pancreatitis), renal disease (pyelonephritis and renal calculi), dermatologic disease (pruritic urticarial papules and plaques of pregnancy, atopic eruption of pregnancy, pemphigoid gestationis, intrahepatic cholestasis of pregnancy), and sepsis. Students were expected to complete a quiz prior to the scheduled simulation activity to assess baseline knowledge of these conditions and build on other pregnancy conditions that had been previously addressed in the term such as hypertensive disorders, bleeding, infections, and preterm labor.

**Figure 1:**
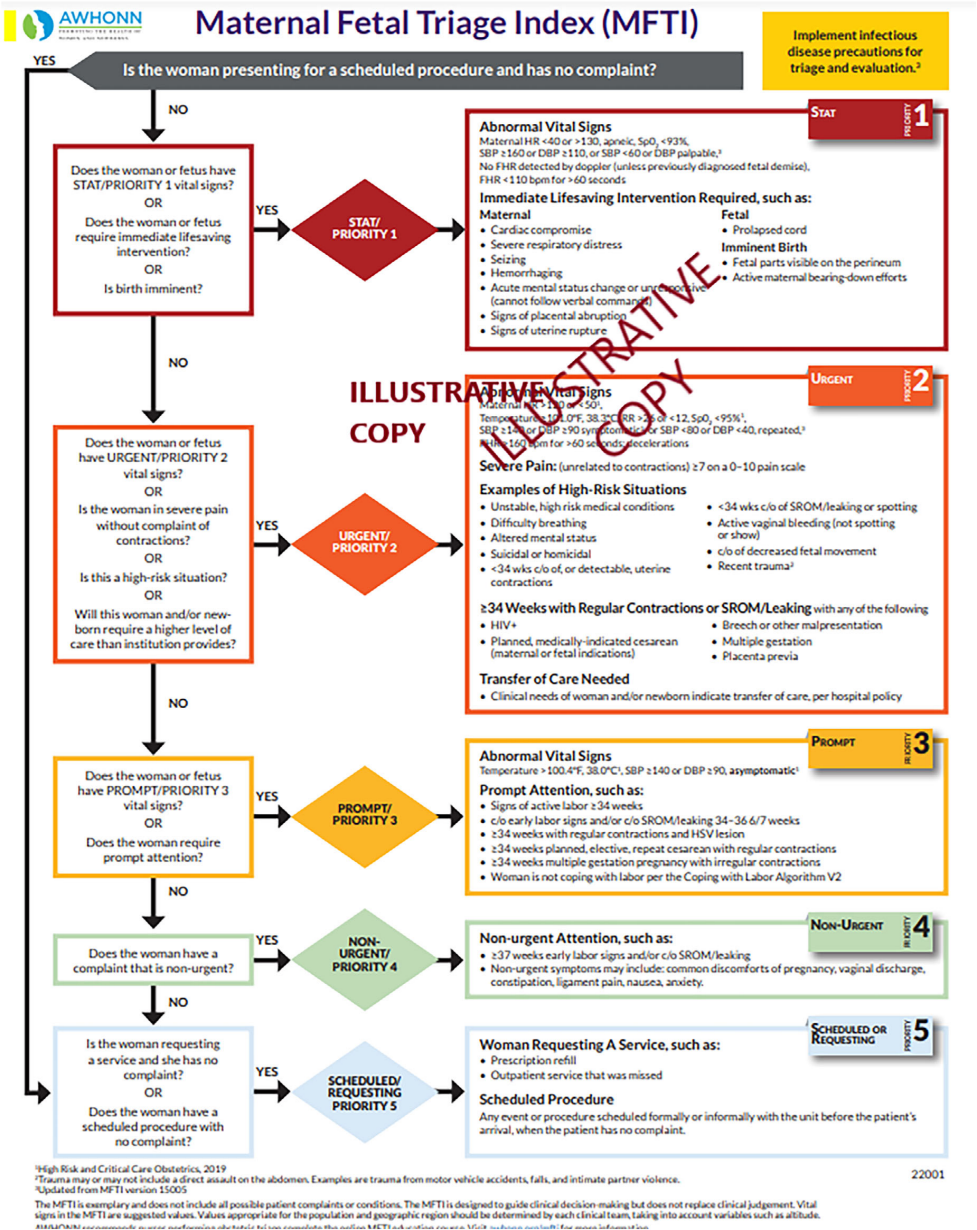
Maternal Fetal Triage Index, 2022 Source: AWHONN Maternal Fetal Triage Index, 2022. Reprinted with permission from the Association of Women's Health, Obstetric and Neonatal Nurses (AWHONN). To access the full MFTI algorithm file, visit www.awhonn.org and the MFTI Online Education Module.

In the required asynchronous video students learned the process of triaging pregnant patients, which included an initial evaluation to determine the acuity and stability of the pregnant patient and fetus. The Maternal Fetal Triage Index (MFTI) tool was used as a template for this acuity assessment (Figure 1), as use of this validated tool improves the quality and efficiency in OB triage settings.^2^, ^8^ Students were provided guidance on using the MFTI to assign priority and determine an appropriate plan of care. The video included content on decision‐making regarding delegation, consultation, referral, and the process of transferring patients to a different facility or higher level of care if necessary.

Preparation for the triage simulation activity occurred throughout the term as students were introduced to the process of evaluating pregnant people through sample cases that were integrated during live class sessions. Introducing this type of learning modality prior to the triage activity helped prepare students on what to expect and reduced anxiety, with the goal of enhancing the triage simulation experience for students.^10^

In Zoom breakout rooms, groups participated in the same set of scenarios; telephone triage, office‐based triage, and hospital‐based triage. Students rotated through the different roles in each of the scenarios. The student provider received a patient summary that included a basic summary of the presenting patients. The student nurse received a script with additional information, including vital signs, recent pertinent laboratories, and fetal monitoring information (Table 1). The student consultant received detailed information about the cases, including differential diagnoses, physical examination findings, and suggested laboratories and follow‐up. The faculty then enabled a dynamic PowerPoint, which allowed students to navigate through different slides based on which room they chose. Students started the simulation in the main triage and selected which patient room to enter into based on the priority assigned by the student provider. The student delegated tasks, assessed the pregnant person, and consulted as appropriate. Although the student provider was able to describe the physical examination skills required and recommend further evaluation based on information received from the student nurse, the activity does not allow for the opportunity to directly evaluate physical examination skills.

**Table 1:** Student Provider Information for the Office‐Based Scenario

Room	Age, y	G/P	Gestational Age	Chief Complaint	Notes
1	31	G2P0101	35 wk 4 d	Swollen feet, a headache and seeing spots	cHTN: no current medications; blood pressure is 150/110
2	26	G2P2002	Postpartum	Lower abdominal pain, heavy bleeding at 2.5 weeks PP	T 38.3 °C (101 °F), P 98, RR 18, BP 102/60
3	38	G3P0020	5 wk	Spotting/cramping	2 prior SABs
4	17	G1P0000	26 wk 0 d	Late to care; reports fatigue and shortness of breath	First prenatal visit; follow‐up to ED visit; given iron and told to follow‐up in clinic

Abbreviations: BP blood pressure; cHTN chronic hypertension; ED emergency department; G/P, gravida/para; P, pulse; PP postpartum; RR, respiratory rate; T temperature; TPAL, Term, preterm, abortion, living; SAB, spontaneous abortion; VS, vital signs.

After each 20‐minute scenario, the small groups came together for a 20‐minute debrief of the activity, and the clinical situations were reviewed. The students discussed the experience, including delegation and acting in different roles, the clinical situations, and what challenges arose during the activity. Debriefing created a space for reflective analysis and digesting of the learning that occurred.^17^ The lead facilitator guided the debrief, answered questions or concerns about the scenarios and roles, and helped prepare students for the next scenario.

## LESSONS LEARNED

This triage activity started as simple online breakout groups with clinical scenarios that were created and based on the experiences of a seasoned midwife faculty member. The scenarios allowed students to work through difficult triage cases; however, the static space did not allow them to simulate prioritization, evaluation, delegation, and consultation. The experience then evolved into an experiential‐based simulation activity using a dynamic slide deck to work through hospital‐based triage patients. The scenarios presented to the students were often challenging and unfamiliar as the students in this course had only attended clinicals in the ambulatory setting. The activity evolved to include scenarios in the ambulatory setting, both telephone and office‐based; the last scenario was an introduction into hospital‐based triage. The scenarios continued to evolve each term to include emerging evidence‐based recommendations, content that students have struggled with throughout the term, as well as low‐occurring but high‐acuity conditions.

The ideal group size and faculty student ratio has also evolved with the development of the activity. Smaller groups with a student provider and a nurse created scenarios where the faculty function as both patient and consultant, which limited the development of interprofessional skills and communication and limited the students’ abstract conceptualization during the activity.^9^ Larger groups allowed for 4 rotating assignments (2 student providers, a nurse, and a consultant), with faculty functioning solely as the pregnant person. Although this fostered collaboration, it limited the experiential learning space for some of the students. When faculty and student ratios permitted, the ideal group size was 3 students and one faculty. The students had the opportunity to rotate through each of the roles: provider, nurse, and consultant. The faculty ensured the activity was flexible, and group numbers could be adjusted depending on resources.

The inclusion of the student as a consultant in the groups simulated an interprofessional space and encouraged students to use available resources and refine communication skills. Interprofessional communication is important for providing optimal patient care as well as creating an environment that fosters the shared responsibility for outcomes.^18^ For example, in the hospital‐based scenarios, a pregnant person presenting with third‐trimester bleeding consistent with a placenta previa or abruption would need a transfer of care by the student provider to the obstetrician on labor and birth; the patient would then prepared for an urgent cesarean birth.

### Curriculum Evaluation and Next Steps

Postactivity evaluations have indicated favorable student responses to the simulation. One student shared the following feedback:

…it's very hard to jump into triage mode when you've never had any experience. I feel like we learned the basics of things like cardiomyopathy, appendicitis, cholestasis, asthma, etc. However, I felt like we were expected to know how a system runs on triage and if we've never seen it or tried it, it's very difficult to have an idea of what to do.

Another commented:

The simulation was great. I feel it made sense while covering the triage topic. It forced us to critically think which was much appreciated. Honestly, it also helped us to know what we need to review as an APRN related to assessments and labs. It helped to know where the gaps are in what we learned this term which can help us to know what we need to go back and review.

Upon completion of this course, NM students’ progress into their next clinical course focused on labor, birth, postpartum, and newborn care where they engage in on‐call phone triage simulations with faculty support. Given the recent success of the triage activity in the *Complex Care of Pregnancy* course, plans are underway to expand simulated triage activities into courses in the program.

## CONCLUSION

This innovative triage simulation activity created an effective way for students to improve knowledge and practice delegation, communication, and consultation while gaining self‐confidence. The activity continues to evolve with the lessons learned; however, the goal of the activity remains the same, to foster a safe environment for midwifery and WHNP students to learn about and practice critical triage skills. The simulation design effectively addresses the challenge of distance‐based education by incorporating both asynchronous and synchronous components while using multiple modalities (telehealth, office, and hospital settings) to prepare students for various clinical situations. The debriefing component is particularly crucial because it allows students to reflect on their performance, receive immediate feedback, and integrate new knowledge into their existing clinical framework, supporting both constructivist and experiential learning theory principles.

This triage simulation experience can be modified and adapted to be used for students at different points in their education and across in‐person, hybrid, and distance‐based programs. It is also important to note that this activity can be tailored to reinforce content that faculty have identified specific cohorts are struggling with or to address conditions that commonly present in certain geographic areas, or to increase students’ exposure to emerging illnesses. Future scenarios should also continue to incorporate concepts of health equity, social determinants of health, and facilitation of care beyond the acute need that often occurs in the hospital‐based triage setting.

## CONFLICT OF INTEREST

The authors have no conflicts of interest to disclose.
